# Understanding the lived experiences of family caregivers of individuals with dementia in Soweto, a South African Township

**DOI:** 10.1177/14713012221118441

**Published:** 2022-08-13

**Authors:** Aqeela Mahomed, Chrisma Pretorius

**Affiliations:** 26697 Stellenbosch University, Stellenbosch, South Africa

**Keywords:** Dementia, mental health, caregiver grief, low-income community

## Abstract

This study was undertaken to understand South African family caregivers’ lived experiences of individuals living with dementia in a predominantly Black African township. A homogenous sample of thirty family caregivers was recruited using purposeful sampling methods and interviewed using a semi-structured approach. Reflective Thematic Analysis (RTA) yielded four broad themes: *Understanding Dementia, Struggles and Sacrifice, Mental Health and Protective Factors.* Findings reflect how dementia is understood by family caregivers and their community, the struggles and sacrifices that they endure, the impact of caregiving on caregiver mental health and the protective factors that enable caregivers to cope, despite their difficulties. Our findings lead to new insights regarding dementia caregiving amongst family caregivers in South Africa. First, there appears to be a shift in perception – away from a cultural/spiritual paradigm – and a lack of pressure to conform to community conceptualizations of dementia among individual caregivers. Second, dementia caregiving had a negative effect on caregiver mental health and elicited stress, anxiety and grief reactions. Third, caregivers did not feel emotionally supported and expressed not receiving any assistance with their daily practical tasks. Fourth, before receiving a diagnosis, family caregivers were viewed as the perpetrators of abuse and/or neglect against their family members with dementia, instead of individuals with dementia being stigmatized by the community due to their behavioural symptoms. Additionally, help-seeking was not hindered by fear or stigma, but was motivated by caregiver distress as dementia-related behaviours began to manifest and caregivers feared being perceived as perpetrators of abuse. Psychoeducational interventions should be tailored to targeted population groups that are in need of further training to address the lack of awareness in communities, insufficient knowledge of dementia amongst healthcare professionals and the practical, emotional and psychological difficulties that family caregivers endure to facilitate mental health care and resilience.

## Introduction

The term dementia characterizes a cluster of neurodegenerative diseases (Devinsky & D’esposito, 2004; Lezak et al., 2012) with high global prevalence rates that are expected to increase considerably by the year 2050 (Morgan et al., 2011; Nichols et al., 2019). Globally, an estimated 55.2 million individuals live with dementia – 1.9 million of which reside in the African region (World Health Organization; WHO, 2021). According to the Global Burden of Disease (GBD) study, the estimated global mortality rate in 2019 was 1.62 million. Furthermore, projected dementia prevalence rates amongst low and middle-income countries such as Sub-Saharan Africa are a reason for concern, as figures are predicted to rise by 70–90% (De Jager et al., 2015). The most common neurodegenerative disorder amongst the dementia cluster is Alzheimer’s disease (AD), which encompasses 60–80% of dementia diagnoses (Alzheimer’s Association, 2017; Kabir et al., 2020; Slattum et al., 2014). Dementia in South Africa is considered as part of the top 50 causes of mortality and is rated 31 amongst global mortality estimates (Meyer et al., 2016).

Despite these increasing prevalence rates of dementia in low and middle-income countries, health services cannot meet the demands that the disease imposes (Kalula & Petros, 2011). Subsequently, family caregivers are expected to provide care for their family members with dementia by tending to their personal (bathing, dressing, feeding, toileting, grooming), medical (administering medication and other healthcare needs such as comorbid illnesses), domestic (laundry and meal preparation), financial and transportation needs (Black et al., 2013; Brodaty & Donkin, 2009; Hooyman & Kiyak, 2002; Joubert, 2005). As dementia continues to manifest and their family member with dementia deteriorates both cognitively and behaviourally (Devinsky & D’esposito, 2004; Dong et al., 2012), caregiving demands increase in intensity, time and complexity (Black et al., 2013; Tremont, 2011). In turn, caregivers are riddled with challenges that affect their physical, psychological, emotional health – factors that have been attributed to caregiver burden (Brodaty & Donkin, 2009; Harmell et al., 2011; Kabir et al., 2020; Prorok et al., 2013; Ransmayr et al., 2018; Sanders et al., 2008; Takai et al., 2011; Truzzi et al., 2012).

Studies have also found that caregivers with depression and higher levels of burden especially nearing the end-of-life stage, are vulnerable to grief reactions that are likely to persist over an extended 12-month period after their family member’s death, thereby meeting an important criterion for a bereavement disorder such as Prolonged Grief Disorder (PGD) or Persistent Complex Bereavement in the Diagnostic and Statistical Manual of Mental Disorders (Fifth Edition) (DSM-V) (Passoni et al., 2015; Tremont, 2011). Caregiver grief has been described by Doka (2004) as ‘the constant yet hidden companion of Alzheimer’s disease and other related dementias’ (p. 140), implying the concept of *dual dying*, where the caregiver first mourns the perceived loss of the individual with dementia before their death (due to their cognitive and behavioural deterioration) and then grieves the loss of the person living with dementia after their physical death has occurred (Moore et al., 2020; Sanders et al., 2008).

The psychological and emotional difficulties experienced by caregivers have also drawn attention in the literature due to its links to caregiver burden. Caregivers have reported decreased well-being, higher stress levels, anxiety and mood disorders (Abreu et al., 2018; Basu & Mukhopadhyay, 2019; Krutter et al., 2020; Tremont, 2011). Moreover, as cognitive and behavioural changes of the individual with dementia manifest (Devinsky & D’esposito, 2004; Dong et al., 2012), caregivers experience feelings of denial and shame and face social exclusion within their communities (Graham et al., 2003; Kahn et al., 2016; Mukadam & Livingston, 2012). Literature has shown that the stigma towards people living with dementia and their caregivers is prevalent across multiple settings, amongst diverse populations and contributes to the burden of care (Blay & Toledo Pisa, 2010; Cheng et al., 2011; Fernando et al., 2010; Gomez-Gomez et al., 2018; Kehoua etal., 2019; Mackenzie, 2006; Mukadam & Livingston, 2012; Su & Chang, 2020). Furthermore, the cultural belief system shared within the community about AD, and related dementias contribute significantly to how people living with dementia and their caregivers are perceived and subsequently treated (Hindley et al., 2016; Mkhonto & Hanssen, 2018; Mukadam & Livingston, 2012).

Cultural belief systems amongst African and minority ethnic communities share the understanding that the symptoms of dementia are consequences of supernatural phenomena such as evil spirit possession, witchcraft or a form of retribution by God (Hussain, 2001; Jett, 2006; Khonje et al., 2015; Mkhonto & Hanssen, 2018; Mukadam & Livingston, 2012; Mushi, 2014). Research conducted in South Africa have all highlighted the presence of indigenous/spiritual beliefs as an explanatory model for dementia in multiple low-income, rural and community settings (Bosch, 2014; De Jager et al., 2015, 2017; Ferreira & Makoni, 2002; Gurayah, 2015; Kalula & Petros, 2011; Khonje et al., 2015; Mkhonto & Hanssen, 2018). In keeping with cultural beliefs about dementia, people with dementia are presumed to be witches and their caregivers held responsible for their family member with dementia’s condition due to their lack of faith and are subsequently shunned by the community (Cipriani & Borin, 2014; Mkhonto & Hanssen, 2018). Furthermore, people with dementia who are considered witches are targets of violent and abusive acts that have resulted in murder (Khonje et al., 2015; Leff, 2014; Mkhonto & Hanssen, 2018). According to the South African Pagan Rights Alliance (SAPRA, 2015), there were 76 witch-killings between 2010–2015 – although this may not reflect the full extent of the problem due to the probability of more cases being underreported (Ferreira & Kalula, 2009; Mkhonto & Hanssen, 2018).

As a protective gesture, family caregivers feel obliged to hide the person with dementia away and refrain from seeking further assistance (Forbat, 2003). In turn, this increases caregiver stress, hinders help-seeking, delays diagnosis and deters service use (Ally, 2009; Cipriani & Borin, 2014; Mukadam et al., 2011; Mukadam & Livingston, 2012; Perry-Young et al., 2018; Robinson et al., 2005).

The largest prevalence of informal caregivers are amongst the Black South African population – ‘double the rate’ (Joubert, 2005, p. 107) across Black African, Coloured, Indian and White population groups.^1^ South African studies that have focused on the dementia caregiver experience highlighted high levels of caregiver burden, resource constraints, the lack of services that caregivers identified in dealing with their daily challenges and the need to intervene with both caregivers and diagnosed family members to facilitate improved overall functioning (Bosch, 2014; Gurayah, 2015; Hendriks-Lalla & Pretorius, 2018; Mahomed & Pretorius, 2020; Pretorius et al., 2009; Van der Poel & Pretorius, 2009). This, together with findings from a sizeable prevalence study (N = 1394) conducted in rural Eastern Cape by De Jager and colleagues in 2017, which indicated a ‘higher than expected burden of dementia in South Africa’ (p. 1094), renders dementia care in South Africa a priority area for individuals with dementia and their caregivers. Despite the multifactorial nature of dementia caregiving and the commonalities found across the literature, a systematic review of 102 qualitative studies of individuals with dementia and their caregivers in communities posit that ‘beliefs and experiences were context-specific and could be polarised’ (Bunn et al., 2012, pg. 7). In light of this, and the additional challenges that a low socioeconomic context, such as a township^2^ may endure in terms of socioeconomic deprivation, resource constraints, poverty, low levels of education and income, social support and the lack of transportation (Kalula & Petros, 2011; Visser & Moleko, 2012) may give rise to specific experiences of South African family caregivers. This is the first South African study that aims to gain both a broader and deeper understanding of the specific experiences of family caregivers of individuals with dementia within a low-income Black African township in SA.

## Methods

### Participants and recruitment strategy

This study recruited thirty Black South African family caregivers of individuals with dementia via purposive sampling methods. Inclusion criteria for the study were (1) participants 18 years and older, English-speaking, family members who reside in the same home as the person living with dementia in the Soweto area and responsible for their activities of daily living (ADLs) for at least one year. Ethical clearance was obtained by the Research Ethics Committee at Stellenbosch University before the study commenced (PSY-2019-10582). Criteria were shared with the Alzheimer’s South Africa office coordinator, who established initial contact with suitable participants to circumvent any initial resistance and facilitate trust. During this process, participants who indicated their willingness to participate were informed by Alzheimer’s South Africa that the first author would contact them to explain the study and arrange their interview.

### Procedure

Upon receipt of the database from Alzheimer’s SA, participants were contacted telephonically by the first author to explain the study’s research objectives. Thereafter, they were invited to participate in the research and encouraged to ask any initial questions pertaining to the study. Participants were informed of their participation as voluntary and that – apart from travel costs to the venue – there will be no costs to them. All participants were reimbursed accordingly for the travel costs that were incurred for transportation to the Alzheimer’s South Africa office. All participants were also informed that they could choose to leave the study at any time and that their withdrawal will not have any negative consequences.

After verbal consent was obtained, interviews were scheduled and conducted by the first author at the Alzheimer’s South Africa office on the date and time best suited to each participant. On the interview day, the research question, aims and objectives were reiterated more comprehensively. Participants were encouraged to ask any additional questions that they had, and the first author addressed any areas of uncertainty. Thereafter, written consent was obtained before data collection commenced. To ensure confidentiality and the protection of the participants’ identities, a numbered system for code names, for example, ‘Family Caregiver 1’ (FC1), was used instead of using any real identifying data. Any information that leads to the revelation of their true identity was omitted. Sample characteristics are outlined in Table 1.

**Table 1.** Sample characteristics.

FC	Gender	Age	Caregiving Dyad	Duration of Caregiving (years)	HLOE
**1**	Female	30	Granddaughter caring for grandmother	3	Tertiary
**2**	Female	50	Daughter caring for mother	1	Tertiary
**3**	Male	28	Son caring for mother	1	Matric
**4**	Male	58	Husband caring for wife	5	Grade 7
**5**	Female	23	Daughter caring for mother	2	Tertiary
**6**	Female	54	Daughter caring for mother	5	Grade 11
**7**	Female	39	Daughter caring for mother	6	Tertiary
**8**	Female	41	Daughter caring for mother	3	Tertiary
**9**	Female	47	Daughter caring for mother	2	Tertiary
**10**	Female	58	Daughter caring for mother	2	Grade 10
**11**	Female	66	Wife caring for husband	3	Tertiary
**12**	Male	47	Son caring for mother	2	Matric
**13**	Female	20	Granddaughter caring for grandmother	2	Matric
**14**	Male	22	Grandson caring for grandmother	2	Matric
**15**	Female	51	Daughter caring for mother	3	Tertiary
**16**	Male	30	Son caring for mother	6	Matric
**17**	Female	52	Daughter caring for mother	5	Matric
**18**	Male	51	Son caring for mother	5	Grade 11
**19**	Female	74	Wife caring for husband	4	Tertiary
**20**	Female	40	Daughter caring for mother	1	Grade 10
**21**	Female	49	Daughter caring for mother	2	Grade 11
**22**	Male	74	Husband caring for wife	2	Tertiary
**23**	Female	54	Daughter caring for mother	4	Tertiary
**24**	Female	60	Sister caring for sibling	3	Tertiary
**25**	Female	72	Wife caring for husband	1.5	Grade 9
**26**	Female	50	Daughter caring for mother	5	Matric
**27**	Male	54	Son caring for father	4	Grade 11
**28**	Female	63	Wife caring for husband	1	Grade 10
**29**	Male	40	Son caring for mother	1	Matric
**30**	Female	64	Wife caring for husband	3	Tertiary

Note: FC = Family Caregiver; HLOE = Highest level of education; Grade = level at school; Matric = refers to graduating from high school in South Africa; Tertiary = refers to college education

### Data collection

Data collection involved in-person, individual interviews^3^ using the semi-structured interview guide in Table 2. A semi-structured interview guide allowed for flexible and interactive engagement with participants as they were able to communicate the meaning of their responses while the researcher clarified using reflective listening. Hence, the questions were made more suitable to the participant (Willig, 2008). The interview guide also included questions that elicited a description of participants’ experiences associated with their role as caregivers. Furthermore, prompting was used where necessary to explore the nuances of their subjective experiences and gain a more in-depth understanding. The duration of each interview was approximately 60–90 minutes. Consent was obtained from participants to audio record their interviews to allow for verbatim transcription. Interviews were transcribed by a transcription service – due to time restrictions and to ensure objectivity and accuracy of participants’ narratives.

**Table 2.** Semi-Structured Interview Guide.

1. How long have you been taking care of your loved one with dementia for?
2. Please help me understand what it has been like for you to live with and take care of someone with dementia.
3. What are your understandings about dementia?
4. How has it been for you in the community since your loved one got diagnosed with dementia?
5. What does your culture say about dementia?
6. Since your loved one was diagnosed, has anything in your life or family’s life changed? If yes, how so?

### Data analysis

Reflexive Thematic Analysis (RTA) was employed to analyze the dataset (Braun & Clarke (2006; 2019). RTA consists of six stages, namely, (*1) familiarization with the data,* (*2) generating initial codes,* (*3) generating themes,* (*4) reviewing potential themes,* (*5) defining and naming themes,* (*6) producing the report.* The process is summarized in Table 3. Of note, an inductive approach to coding was used to ensure that the codes best represent the content of the data and are not derived to suit a ‘pre-existing coding frame’ (Byrne, 2021, p. 7).

**Table 3.** Data Analysis Process.

Stage	Analysis Process
Familiarization with the data	The first author immersed herself into the data by reading through the transcripts while simultaneously listening to the audio to ensure the accuracy of the transcriptions. This enabled the author to recall the atmosphere of the interview and note any reflections or observations that were identified. Each new reading and listening to the audio allowed for new insights.
Generating initial codes	Rigorous notes were produced that represented the transcripts and audio recordings. Initial codes were produced to organize the data into meaningful units based on the details provided by the participant. Qualitative data analysis software – ATLAS.ti – was used due to the copious amount of data, to facilitate this step and assist with the thematic process. An inductive approach was used to generate codes that reflected the content of the data.
Generating initial themes	Using ATLAS.ti, the identified codes were categorized into themes based on shared associations. Each category was assigned a descriptive label and the meanings of and relationships between codes were interpreted.
Reviewing potential themes	Patterns across the coded data were distinguished and the entire data set was reviewed. Overlapping themes were collapsed and refined.
Defining and naming themes	A list of major themes and subthemes were categorized and the narrative of the themes were identified and conceptualized within the broader data set in response to the research questions.
Producing the report	A narrative account of participant data was presented using short excerpts from the transcripts to convey our salient findings in response to our research questions.

### Trustworthiness

The primary aim of trustworthiness is to evaluate the procedures employed during data analysis to ensure that findings convey the closest meanings elicited from the caregivers (Lietz et al., 2006). The proposed study used three methods to ensure the trustworthiness of its findings: *Reflection* plays a crucial role in qualitative research (Long & Johnson, 2000). During reflection, the author not only examines the thoughts of their research participants but is mindful of their own. A journal to enable author self-reflection was kept for the duration of the interview process. Information from all interviews and journal material was discussed between co-authors regularly. Thus, ensuring that subjective biases do not influence how the data was interpreted, allowing for objective analysis. *Response validation* is vital to ensure that the collected information accurately reflects the intended message (Long & Johnson, 2000). Hence, during and after each interview, the first author allowed participants to confirm the meanings they conveyed. The first author reiterated participant responses and they were given the opportunity to confirm that the meaning of their responses was accurately interpreted. The aim of this was to establish an accurate and clear understanding of the experiences of the participants. *Peer debriefing* occurs by discussing and verifying the data with colleagues on an ongoing basis to consider additional and alternative perspectives for the duration of the entire interview process (Long & Johnson, 2000). The interviews, themes and interpretations thereof were discussed between authors throughout the process.

### Findings

The data analysis process yielded four broad themes: *Understanding Dementia, Struggles and Sacrifice, Mental Health and Protective Factors.* Within these broader themes, subthemes were generated to elucidate the complexities of family caregivers' lived experiences. Selected quotes are presented with participant characteristics to illustrate themes. A tabulated overview of illustrative quotes is available as supplemental material.

## Theme 1: Understanding dementia

### Perceptions

#### Spiritual

Most caregivers described behavioural changes displayed by ‘grannies’ such as ‘going in the open naked’ or ‘wak[ing] up without wearing clothes’, ‘seeing things’, accusing their children of stealing their money in the home and being found alone in other community members back yards at night while talking in a way that does not make sense. Furthermore, caregivers explained that the Black African community perceives these behaviours as demon possession, an ancestral calling or that the person displaying such symptoms is a ‘witch’ who ‘bewitch others’ (Q1–Q3).^4^ Notably, none of the caregivers explicitly conceded with any of the cultural perceptions ascribed to their family members with dementia’s observed behaviours. Some caregivers expressed the sentiments: ‘no magic, no witchcraft’, ‘I don’t believe in witchcraft’.

‘In my culture…you find that there are aunties, old ladies who’ve been banned, who’ve been characterized no, who’ve been called witches and they either they were killed, they were stoned, they were banned because they bewitch others and others will be going in the open naked and then they will be branded witches yes’ (Q3 – 47 year old, daughter caring for mother)

#### Ageing and forgetting

Most caregivers expressed that their community commonly perceives memory loss as a natural aspect of ageing. Hence, when their family members with dementia are ‘forgetful’, it is considered ‘normal’ due to their age. Some caregivers’ perceptions aligned with this notion as they mentioned that their family members with dementia are ‘not sick’. In contrast, other caregivers characterized dementia by one of its hallmark features – ‘a loss of memory’ (Q4–Q6).

‘I think us in the more urban areas we know they call it like an old people's disease. So they don't really define it as dementia. They just say aaah it's normal. She's old’ (Q4 – 51 year old, son caring for mother)

#### Medical illness

Some caregivers ascribed the behavioural changes that their family members with dementia displayed to a medical illness or disease. Caregivers were unable to provide any further information regarding the nature of the disease, its process and its progression. These caregivers consider dementia as any other medical illness such as ‘TB’ or ‘diabetes’ and that the ‘brain diminishes’ (Q7–Q10). Notably, most of these caregivers mentioned having reached this understanding after obtaining more information through their research and the efforts of their community representative from Alzheimer’s SA. In contrast, one caregiver explicitly stated ‘I wouldn’t say it’s a medical sickness because there is no medicine for it. It cannot be cured’.

‘The way I make sense of it is it is what it is. People get sick, others have cancer and my mum has dementia or others have TB or others have diabetes. It just is what it is’ (Q7 – 40 year old, daughter caring for mother)

‘I think Dementia is another illness that what it just happens or it’s hereditary’ (Q8 – 54 year old, daughter caring for mother)

#### Going mad

Very few caregivers mentioned that their family members with dementia might be perceived as ‘mad’ and/or ‘crazy’ by community members. One of these caregivers emphasized how they were told explicitly by community members that their family member with dementia was ‘insane’ before receiving a diagnosis (Q12) and how the stigma abated once people had a better understanding (Q13).

‘Ja before from people who actually who told us like she is… I think… insane’ (Q12 – 50 year old, daughter caring for mother)

‘You know when my mom is coming, if she's angry, she will bang the door, bang the gate, everything, going, passing through all the people and if you are angry it’s not easy for you. You know we were like a laughing stock. But now people, as they know now she is, she has a dementia. They appreciate, you know, and they understand’ (Q13 – 39 year old, daughter caring for mother)

### Challenges

#### Awareness, receiving a diagnosis and accessing treatment

A majority of the caregivers stated that they had no prior awareness of dementia or its symptoms. Hence, most caregivers described the challenges that occurred due to their lack of awareness at the outset. Firstly, when caregivers began to notice the marked behavioural changes of their family members with dementia, they struggled to make sense of what had been occurring (Q14–Q15) and decided to take their family member with dementia to their local clinic to access treatment. Secondly, upon arriving at the clinic, caregivers expressed their frustrations at the healthcare professionals that they engaged with who ‘didn’t know’ and ‘dismissed [the caregiver] just say[ing] it is old age’ (Q16).

‘You know what? Before, before I did not understand it, yes, I was like that, because of I used to shout at her even. Why doing this? Why are you doing that?’ (Q15 – 51 year old, daughter caring for mother)

‘From reading, you know what I did in 2016 I took her to one of our local clinics and I said to the doctor it was for something else but I asked the doctor to help me I said this is what I see my mom is doing, this she’s going through this, she say don’t you know it is old age but then my mother knew that there was such an illness you know and the doctors just let me go dismissed me and just said it is old age’. (Q16 – 58 year old, daughter caring for mother)

At this juncture, several caregivers voiced how their struggles were exacerbated because they were unable to explain to healthcare professionals what their family member with dementia was experiencing – creating a cycle of uncertainty and helplessness (Q17–19). Thirdly and consequently, these individuals did not receive a diagnosis – timeously or at all – and could not access any treatment or assistance for either the caregiver or their family member with dementia (Q20).

‘I think at first, because we didn’t know. So in the beginning, because we didn’t know, we were left to our assumptions. Because obviously, even though we had questions, but we’re also African. So when you go to places where medical practitioners are supposed to know, and then they’re saying they don’t know, but now we know that they didn’t know because they were not psychiatrists or mental doctors in a sense’ (Q17– 40 year old, son caring for mother)

‘Okay. That’s when we say “okay, she’s not okay” and then we went to the Clinic but still it took time because you know clinics, they didn't like transfer us to the doctor that she is in right now. It took us surely a year doing the same thing over and over again. Just to get treatment after being diagnosed, just to get treatment’ (Q20 – 47 year old, daughter caring for mother)

#### Community awareness

Some caregivers expressed how the lack of awareness within the community also proved challenging initially, as neighbours began to question the behaviours of these individuals and the stories that they were telling. Caregivers were accused of neglecting, mistreating and stealing money from their family members with dementia. This appears to have alerted caregivers to the gravity of the situation, which prompted professional help-seeking (Q21–Q22).

‘Yes because I was tired not knowing what to do, because if it was so worse this time, because she was fighting and going outside and crying to other people, people they will come to me and say your mother is always crying. She is saying that you don't take care of her, and I would say I don't understand. And then your mother say that your children they, they are, they give her a problem. So it becomes something that now, it's like, we are treating her bad. And people now are becoming involved you know. And me I was trying, telling myself that one day she is going to be all right as long as we can keep on doing what is right towards her, but no. Nothing had helped, nothing, nothing you can do whatever you know to do, you can try to understand but you will never understand her. So I saw it becoming worse now as she is going outside crying to other people, it is worse. So I have just decided that I should take the police and take her to hospital’ (Q22 – 52 year old, daughter caring for mother)

### Enduring ambiguity

This subtheme is presented independently as it permeates all other themes and subthemes. It reflected the current perceptions of caregivers, appeared to be an ongoing challenge and contributed to the difficulties that caregivers were still experiencing. Thus, it shaped the overall narrative of the caregiver experience. All caregivers were asked to articulate their understanding of dementia as their familiarity and caregiving experience grew over time. Notably, most caregivers were still struggling to make sense of dementia and its manifestations despite receiving a diagnosis and accessing the help available to them (Q23–26). Although some caregivers illustrated their progress after obtaining more information – both as a result of their research and the educational efforts of Alzheimer’s South Africa, there was still a sense of ambiguity reflected in their perspectives of dementia (Q27–Q29).

‘To be serious … to be honest, I don't understand dementia’ (Q23 – 30 year old, son caring for mother)

‘Look, how I make sense of them is – you see my daughter over here – one thing I’m seeing is that there are habits in how we remember and we recall and remember anyway. And I see those habits in us, all of us, so I think it’s, I don’t want to call it dementia, but it’s just a habit in recalling and thinking. So I’ve just seen that with her it’s gotten to like a scary place, I’ll call it that’ (Q25 – 54 year old, daughter caring for mother)

‘Because since she got Alzheimer’s we don’t know what’s happening in her brain and this time she is worse. And now she’s worse, worse. I don’t know she’s maybe in which stage. I know there’s 5 stages, I don’t know. You tell her something now, 2 seconds she forgot. 2 Seconds she forgot’ (Q28 – 74 year old, husband caring for wife)

## Theme 2: Struggles and sacrifice

### Personal sacrifices

More than half of the caregivers explained how taking care of their family member with dementia changed their lives and the sacrifices they chose to make due to their family member with dementia’s illness. Specifically, caregivers decided to either leave their jobs (Q30–Q32) or put their studies, dreams and ambitions aside to better ‘understand’ and tend to the needs of their family members with dementia (Q33–Q34).

‘Well I had to, I had to leave my job five years ago decide ja to say I’ll stay with this woman to understand what is it that she’s going through before I can bring in someone you know who would be our help yes’ (Q32 – 49 year old, daughter caring for mother)

### Caregiver roles

Some caregivers, most of whom were taking care of their parent with dementia, alluded to a ‘role reversal’ since assuming caregiving responsibility of their family member with dementia (Q35) and highlighted the difficulty of this experience as it created conflict between the dyad (Q36). Furthermore, caregivers voiced their struggles regarding the pressures of fulfilling multiple caregiver roles, such as a mother taking care of her children (Q37–Q38), a sister taking care of her siblings (Q39) and a grandmother taking care of her grandchild with epilepsy (Q40) while still being expected to take care of the household and their family member with dementia.

‘Whenever I took decisions, she will fight with me saying that I want to be the mother of the house. She is the mother it is so difficult’ (Q36 – 50 year old, daughter caring for mother)

‘Yes, it's because I have children, of which I have to take care of, and eish, yes, as I am single mother, as I am a single mother I need to ensure that my kids, I have to put bread on top of the table nè, yes, and taking care of my mother of which we had ups and downs and no one would understand my problem’ (Q38 – 41 year old, daughter caring for mother)

‘So, you are taking care of mom? You are taking care of the household? Taking care of your siblings? If you do not take care of yourself? You feel like there is no choice?’ (Q39 – 23 year old, daughter caring for mother)

### Lack of support

Few caregivers stated that they had support, mostly from immediate family members, to assist with the practical aspects of caregiving tasks associated with activities of daily living (Q41). Hence, the majority of the caregivers expressed receiving no caregiving support and were left ‘alone’ to take care of their family members with dementia (Q42–44), thereby exacerbating their struggles. Notably, although some caregivers acknowledged receiving emotional support, they either – did not *feel* supported due to differences in opinion, insensitivity of the family as perceived by caregivers – or that emotional support was not what they needed (Q45–47).

‘But sometimes I can feel it because there is nobody to help me. You understand? I am alone. I have to clean. Make sure the house is clean. To cook, to wash, everything is upon my shoulders’ (Q44 – 47 year old, daughter caring for mother)

‘People are not supportive, they just listen to your stuff, that’s the support you get, they just listen, they don’t give you advice on what you should do, how to handle the person what not to do you see, they don’t give you that on how to take care of the situation when it happens, so I won’t say I’ve had support from the outside that much no’ (Q47 – 60 year old, sister caring for sibling)

### Social withdrawal

Majority of the caregivers explained how their lives had changed due to their conscious decisions to withdraw from social engagements (Q48). Caregivers commonly cited feeling guilty (Q49), worried and avoidant in the event of ‘something happen[ing]’ to their family member with dementia in their absence (Q50–51) or having to answer to others who lack understanding about the well-being of their family member with dementia (Q52). Furthermore, some caregivers also verbalized being unable to take their family member with dementia to social events or public places due to their dementia-related behaviours, which in turn, is a ‘stressful’ experience for the caregiver (Q53).

‘So… But I’ve been going out less since she was diagnosed with Alzheimer’s. Again, it comes back to that guilt thing of I’m out here having fun and she’s at home maybe bored or something’ (Q49 – 30 year old, granddaughter caring for grandmother)

‘I wouldn’t go, even when we go the short distance to the families, I must have my gloves, I must have my 2 diapers spare, I must have my wipes, I must have my disposable bags, should we be there for 2, 3 hours, you’re sitting there because you can’t leave him alone. I must carry all those things. And when people go up and down chatting, chatting, I must keep on checking up on him. But no, let me go and check – it’s stressful’ (Q53 – 64 year old, wife caring for husband)

## Theme 3: Mental health

### Psychological distress

When caregivers were asked if and how taking care of their family member with dementia affected their mental health, all of the caregivers in this study reported elements of psychological distress. Most caregivers reported feeling ‘worr[ied]’, ‘sad and fearful’ and ‘anxious’ due to the unpredictability of their family member with dementia’s behaviour, especially at times when the ‘disease has escalated’ and then ‘disappear[s]’ and what this would mean for their future (Q54–Q57). Furthermore, several caregivers expressed feeling ‘frustrated’ (Q58–Q59), especially at times when they were unsure of how to manage their family member with dementia’s challenging behaviours – thus eliciting feelings of helplessness and overwhelm (Q60). While some caregivers described being in denial – both initially (Q61) and at present (Q62), as they struggled to accept what their family member with dementia had to endure, others acknowledged feeling ‘angry’, ‘irritable’ and ‘stressed’ as they struggled to understand and communicate with their family members with dementia (Q63–Q65).

‘Just constantly thinking about it. Constantly thinking about it, constantly worrying about it and thinking about the unknown. How far is it going to go with her, is she going to do the things that we heard that other people do, how long does she have before she leaves us. You know, you just ask yourself about the future that is not yet here, and it’s very, it can drain you mentally’ (Q54 – 52 year old, daughter caring for mother)

‘It’s anxiety more than anything, and it’s anxiety – to be very honest – it’s the anxiety around being able to control the environment. Ja, that’s my thing. Like when I feel it’s outside of my ability to handle, that’s when I get very anxious’ (Q57 – 47 year old, son caring for mother)

‘Ja. I didn’t accept that easily, because you sort of like wanted to… you said, ag, maybe it’s not dementia, maybe it was just something she forgot. You know, stuff like that’ (Q61 – 39 year old, daughter caring for mother)

‘When you try to communicate with them and then they don't respond. So if you don't understand, it will be like … I don't know, you'll be angry’ (Q63 – 51 year old, son caring for mother)

### Caregiver grief

Almost every caregiver mentioned feeling ‘sad’ when prompted about the impact of caring for their family member with dementia on their emotions. After exploring their sadness further, most caregivers described the notion of ‘los[ing] someone while you are still looking at them’ – referring to both the loss of their identity as they ‘slip away every day’ and the loss of their relationships as they ‘miss’ the role that their family member with dementia previously held for their families. Some caregivers articulated this as ‘grieving’, and expressed how ‘this is worse than death’ due to the lack of ‘closure’ as they watch their family member with dementia decline cognitively (Q66–71).

‘So you're grieving the person they used to be because she's definitely not the same person she was before. And you're grieving them in your lives because, you know, they're going to eventually stop talking. They're going to forget who you are. You are used to watching them slip away every day’ (Q67 – 74 year old, husband caring for wife)

‘I always say to people this is worse than death because when a person passes on, you know there’s closure, you bury them and then you deal with the process in terms of grief, so in my case, I’m grieving the loss of my mother even though she’s here’ (Q70 – 58 year old, daughter caring for mother)

## Theme 4: Protective factors

### Coping mechanisms

Half of the caregivers in this study expressed using prayer and the reliance on God to give them strength and understanding to manage their role as caregivers (Q72–Q73), while others described how music and reading helped bring a sense of calmness and clarity that enabled them to cope with their daily challenges (Q74–Q75).

‘It is very difficult. I pray. If I feel, even if my emotions are just down, I just sit down and pray. God, please make understand my mother. Make me, you know because if I cannot, who is going to take care of her? Other people who will not understand her the way I do understand. So, I pray. Yes’ (Q73 – 49 year old, daughter caring for mother)

‘Yes so I think that’s how I calm myself down because if I feel that my mind has started and I don’t understand myself, I usually go the library and then read some books that that are interesting or maybe there is a subject that I want to know about it, the time for me to take that book and sit down and read and take newspaper round and read, that’s how I calm myself down’ (Q75 – 54 year old, son caring father)

### Loyalty, devotion and commitment

Most caregivers expressed their loyalty, devotion and commitment to taking care of their family members with dementia. They believed that no one else would understand and take care ‘properly’ and that they could not ‘throw [them] on the street or take [them] to a home’. Hence, most caregivers took personal responsibility for the protection, care and well-being of their family members with dementia (Q76–Q79).

‘You just tell yourself that it is my mom. I cannot throw her on the street or take her to a home. It is worse there because if I am frustrated what about the people at her room. Maybe they deal with her differently. Ja, so I rather be the one who is frustrated about her with all the family members’ (Q77 – 54 year old, daughter caring for mother)

‘Even if I have a helper sometimes it gets frustrating ‘cos you feel like they don’t know enough to help you. It’s to do things yourself. That’s why I ended up coming to stay with mum, because I found that, you know, there’s little things but they just don’t do it properly. And it just felt like maybe it’s better if I go and do it myself’ (Q78 – 52 year old, daughter caring for mother)

### Strengthened bonds

Some caregivers reflected on how taking care of their family member with dementia strengthened the bond between them. They expressed how – with a better understanding of dementia – they have grown to love the family member that they are taking care of ‘even more’ and ‘unconditionally’ – despite their challenging behaviour (Q80–Q82).

‘…it makes me to love her more. And funny enough, even if she says those things in my absence, when I knock at the door, she’s like, she opens her arms, she wants to hug me and I would, okay, and I would give her a hug and tell her that I love her and I need her and whatever. So, it makes me understand the disease and not the person so that I love her regardless of what she is doing’ (Q80 – 40 year old, daughter caring for mother)

‘So it changed me, because, before I knew the disease, I couldn’t understand, I couldn’t. But now it makes me to love her because I know that she’s sick’ (Q81 – 58 year old, daughter caring for mother)

### Developing resilience and personal growth

Most caregivers explained how they adapt and accept their role as caregivers as their ‘mind adjusts to it’. They stated ‘tak[ing] it as it comes’ and ‘acclimatizing to the situation’ as essential attitudes that have enabled them to better navigate their role as a caregiver (Q83–Q87). Furthermore, most caregivers expressed learning patience, understanding and compassion through their caregiving experiences – signifying personal growth (Q88–90).

‘Now, I think I am kind of getting used to that thing that like he has dementia and needs to be taken care of because he forgets very quick. So, right now I am getting used to it. Yes. I am fine. I think I have adapted to this thing, yes’ (Q83 – 63 year old, wife caring for husband)

‘Oh, I have learned so much. I had to grow very patient, loving… You know you need to be very strong and you need to understand people’ (Q90 – 49 year old, daughter caring for mother)

## Discussion

This study was undertaken to understand South African family caregivers' lived experiences of individuals living with dementia in a predominantly Black African township. To meet this objective, our findings reflect how dementia is understood by family caregivers and their community, the struggles and sacrifices that they endure, the impact of caregiving on caregiver mental health and the protective factors that enable caregivers to cope, despite their difficulties.

Family caregivers highlighted two prominent perceptions that is prevalent within the larger Black African community regarding their family member with dementia’s condition. First, consistent with other South African research in underprivileged communities, psychiatric behaviours such as delusions, hallucinations, wandering and socially inappropriate conduct are perceived as spiritual phenomena as per their cultural belief system. Caregivers specified these phenomena as demon possession, ancestral calling or that the person displaying such symptoms is a ‘witch’ who ‘bewitch others’ (Bosch, 2014; De Jager et al., 2015, 2017; Ferreira & Makoni, 2002; Gurayah, 2015; Kalula & Petros, 2011; Khonje et al., 2015; Mkhonto & Hanssen, 2018). Distinctively, it should be highlighted that most family caregivers in this study did not personally concur with spiritual perceptions held by their community. Some caregivers explicitly expressed the notion ‘no magic, no witchcraft’. This may suggest that caregivers do not feel any pressure to conform to the beliefs held by their larger community. Second, the memory loss that the person exhibits is considered by the larger community as part of the ageing trajectory. Some family caregivers in this study concurred with this community belief while others recognized it as a medical illness. Similar findings were reported in other South African samples (De Jager et al., 2015; Ferreira & Makoni, 2002; Gurayah, 2015) and prevalent among communities both in Sub-Saharan Africa (Mushi et al., 2014; Owokuhaisa et al., 2020) and international samples (Hossain et al., 2020).

Furthermore, an important finding was the ambiguity that family caregivers still held despite their understandings described above (Brooke & Ojo, 2019). Caregivers still struggled to demonstrate a clear understanding of dementia even after receiving a diagnosis and accessing treatment – although some acknowledged progress after obtaining more information through support services such as Alzheimer’s South Africa and their research efforts.

Central challenges experienced by family caregivers related to awareness, diagnosis and accessing treatment. Most caregivers emphasized that their lack of awareness at the outset affected their ability to make sense of their family members with dementia’s condition and access professional help – which included an accurate and/or timely diagnosis, treatment or any support services as reported in several South African studies (De Jager et al., 2015; Ferreira & Makoni, 2002; Gurayah, 2015; Kalula & Petros, 2011; Mahomed & Pretorius, 2020). Notably, caregivers frustrations were exacerbated – both by their struggle to explain to healthcare professionals what they had been experiencing (Mushi et al., 2014) and by the lack of professional knowledge within primary healthcare settings consistent with South African studies (De Jager et al., 2015; Kalula, 2013; Mkhonto & Hanssen, 2018). Some caregivers also highlighted that the lack of dementia awareness in the community often led to accusations of caregivers as the perpetrators of abuse and neglect instead of individuals with dementia being rejected or ostracized by the community due to their behaviours (Khonje et al., 2015). This is an interesting and contrasting finding – despite the cultural perceptions of dementia and the lack of awareness in the community, stigmatizing experiences were minimally reported amongst these caregivers.

Furthermore, help-seeking efforts by caregivers in this sample appeared motivated by the distress of being seen as the perpetrator and/or as a result of their family member with dementia’s strange and unpredictable behaviours. Help-seeking was not hindered in this study by fears that their family member with dementia would be in danger due to the community’s cultural conceptualization of their behaviours as commonly cited in the literature (Kehoua et al., 2019; Mkhonto & Hanssen, 2018).

Additionally, caregivers described the sacrifices and struggles associated with taking care of their family members with dementia and the life changes that this brought in various aspects of their lives. Some caregivers sacrificed their aspirations and ambitions and had to leave their jobs (Omiya et al., 2021; Owokuhaisa et al., 2020), while most caregivers expressed consciously avoiding social engagements due to the guilt, worry and stress of either managing dementia-related behaviours in public or leaving their family member with dementia at home (Brooke & Ojo, 2019; Gurayah, 2015; Khonje et al., 2015).

In keeping with existing literature, most caregivers who were taking care of a parent with dementia mentioned conflict with their family members with dementia since assuming some of their responsibilities such a paying bills and making decisions that affect the household – and noted how their role shifted to a parentified one (Ducharme et al., 2013; Spigelmyer et al., 2018). Furthermore, other caregivers expressed feeling pressured by the multiple roles that they had to fulfil – in addition to their newly acquired role as caregivers – such as mother, sister, grandmother and the household responsibilities that are usually expected to be taken care of by them (Tuomola et al., 2016; Yang et al., 2019).

Caregiver support in the literature has distinguished between ‘instrumental’ (Brodaty et al., 2009, pg. 222) help – referring to daily tasks of caregiving – and emotional support that influence caregiver well-being (Jarrott et al., 2005; Tuomola et al., 2016). Very few caregivers expressed receiving instrumental support, and most felt that they had to take on the role of dementia caregiver alone. Disparate findings were demonstrated amongst South African male caregivers in a low socioeconomic community in the Western Cape, where caregivers were reported to have received informal support from close social networks (Hendricks-Lalla & Pretorius, 2018Hendriks-Lalla & Pretorius, 2018). Notably, some caregivers in this study acknowledged receiving emotional support but did not *feel* supported or received the support they needed. The complexity of this dynamic has been shown to increase caregiver burden in the literature and suggests that informal support may not be as beneficial as presumed (Jarrott et al., 2005; Han et al., 2014).

Findings of this study support the substantial body of research that demonstrate the adverse effects of being a dementia family caregiver on mental health, which encompassed both psychological and emotional functioning (Abreu et al., 2018; Basu & Mukhopadhyay, 2019; Krutter et al., 2020; Song et al., 2018; Vallée & Vallée, 2017; Win et al., 2017; Xiong et al., 2020; Yap et al., 2005). *All* caregivers in this study reported an element of psychological distress associated with their experience of dementia caregiving. They described feeling ‘worried’, ‘sad’, ‘fearful’, ‘anxious’, ‘frustrated’, ‘angry’, ‘irritable’ and ‘stressed’ as caregivers navigated different aspects of their daily caregiver tasks. Caregiver psychological distress centred around strained communication, the management and unpredictability of dementia-related behaviours and denial regarding the disease process that their family members with dementia had to inevitably endure. A prominent finding amongst South African family caregivers in this study was caregiver grief. Most caregivers expressed feeling a particular sadness as they witnessed their family members with dementia deteriorate and experience a loss of identity and meaningful relationships. Caregivers described a process akin to ‘grieving’ that is ‘worse than death’ and that no closure is found because the decline in functioning occurs ‘while you are still looking at them’. The two concepts used to describe this process have been documented in international literature and used interchangeably. These are *anticipatory grief* (Cheung et al., 2018; Holley & Mast, 2009; Lindauer & Harvath, 2014) and *pre-death grief* (Hovland, 2018; Kobiske et al., n.d; Moore et al., 2020), which have been shown to augment psychological and emotional strain.

Despite the challenges, sacrifices, frustrations and ambiguous understandings of dementia as described above, protective factors emerged that appeared to facilitate coping as a dementia caregiver. As described in other studies, caregivers explained the role of religious conviction (Chan et al., 2019; Fife et al., 2020; Teahan et al., 2016), music and reading (Meyer et al., 2015; Schüz et al., 2015) that evoked a sense of calmness and clarity for them.

Furthermore, most caregivers expressed a sense of loyalty, devotion and commitment to their role as caregivers (Lindeza et al., 2020; Navab et al., 2012). They appeared to consider themselves personally responsible for the protection, care and well-being of their family members with dementia (Meyer et al., 2015) due to a lack of trust in anyone else to fulfil the caregiver role (MacLeod et al., 2017). Perhaps this sense of duty is embedded in the concept of Ubuntu (Gurayah, 2015) – an important sentiment within African culture, where human life, respect, dignity, compassion, unity and cohesion is revered (Mugumbate & Andrew, 2013).

On the other hand, it appears that the same sentiment cannot be applied to extended family members and the larger community as most caregivers in this study expressed carrying this weight alone. Despite this, some caregivers experienced a strengthened relationship and unconditional love (Bjørge et al., 2019; Lindeza et al., 2020) as they learn to understand the course of dementia better. Additionally, many caregivers reflected on their ability to adjust and accept their role as they navigate their dementia caregiver experience – facilitating resilience (Polenick et al., 2020; Song et al., 2018). Furthermore, most caregivers noted meaningful qualities that they have begun to acquire, such as patience, understanding and compassion, which suggests personal growth, thereby easing caregiver burden (Teahan et al., 2016).

### Limitations

Caregivers were purposively selected from a homogenous sample from Alzheimer’s South Africa – their voluntary participation assumes a willingness to share their experiences and that they had already been receiving support. However, it should be considered that this study may not have fully captured the experiences of caregivers who were inaccessible (van Wijngaarden et al., 2018) – perhaps indicating unremitting struggles or covert elements of the family caregiver experience that could have been an important area of study.

## Conclusions and recommendations

To our knowledge, this was the first qualitative South African study undertaken to gain a deeper, nuanced understanding of the lived experiences of dementia family caregivers in a low-income Black African community. Our findings highlight and reinforce some of the core daily challenges reported amongst other South African and international samples within their respective contexts. These findings include the lack of awareness of dementia amongst individuals within their communities and the hindrance it poses diagnostically and therapeutically; insufficient knowledge amongst health care professionals that exacerbate the caregiver struggle to access services and support; and the difficulties and frustrations that family caregivers face as they navigate various roles and responsibilities concurrent to dementia caregiving.

Of significance, findings have emerged in this study that lead to new insights regarding dementia caregiving amongst primary caregivers in South Africa. First, there appears to be a shift in perception – away from a cultural/spiritual paradigm – and a lack of pressure to conform to community conceptualizations of dementia among individual family caregivers. Second, as with international samples, dementia caregiving affected caregiver mental health negatively, and evoked stress, anxiety, irritability, fear, worry, anger and sadness. In exploring caregiver sadness, the nature of this sadness emerged as caregiver grief – the process akin to grieving that family caregivers undergo as they watch their family members with dementia deteriorate. An interesting and third finding was the extent to which family caregivers felt supported or received the type of support that were aligned to their needs. Caregivers did not feel emotionally supported and expressed not receiving any assistance with their daily practical demands either. Fourth, before receiving a diagnosis, family caregivers were viewed as the perpetrators of abuse and/or neglect against their family members with dementia, instead of individuals with dementia being stigmatized by the community due to their behavioural symptoms as commonly reported in caregiving literature. Similarly, help-seeking was not hindered by fear or stigma commonly disclosed in dementia caregiving literature, but was motivated by caregiver distress as dementia-related behaviours began to manifest and caregivers feared being perceived as perpetrators of abuse.

In light of this, it is recommended that further research explore these avenues to better inform holistic interventions for caregivers and healthcare professionals that are tailored to the cognitive, practical, psychological and emotional struggles of the dementia caregiver. Psychoeducational interventions are important to consider and tailor to targeted population groups that are in need of further training to address the lack of awareness in communities, insufficient knowledge of dementia amongst healthcare professionals and the difficulties that family caregivers face in their daily caregiving tasks. For example, nurses and health care workers in primary health care settings need rigorous education and training to accurately screen for, manage and refer individuals with dementia and their families to appropriate professionals for diagnostic and treatment purposes. For family caregivers, psychoeducational programmes should be focused on the educational and practical aspects of dementia caregiving, but also empowering caregivers with coping skills to manage their various roles and responsibilities and self-care strategies to facilitate caregiver well-being. It would prove useful to provide practical training to health care workers and volunteers in the community to assist with home-based care, thereby offering respite and alleviating caregiver burden. Additionally, clinical interventions such as support groups, counselling or psychotherapy should be made accessible to families and their family members with dementia. This would serve to provide emotional and psychological support, thereby managing psychological distress, treating caregiver grief and facilitating mental health care and resilience.

## Supplemental Material

Supplemental Material - Understanding the lived experiences of family caregivers of individuals with dementia in Soweto, a South African Township Click here for additional data file. Supplemental Material for Understanding the lived experiences of family caregivers of individuals with dementia in Soweto, a South African Township by Aqeela Mahomed and Chrisma Pretorius in Dementia

## Notes

1. Black, White, Coloured and Indian are the predominant racial categories used to classify different population groups in South Africa imposed by the Apartheid regime (Statistics South Africa, 2012).

2. The term township refers to segregated neighbourhoods that were intentionally designed under colonialism and apartheid, in order to serve as residences for workers from economic centres in the city (Scheba & Turok, 2019).

3. Data collection occurred during 2019, before the emergence of the Covid-19 pandemic.

4. Quote numbers 1 to 90 correspond to comprehensive participant quotes provided as supplemental material.

## References

[bibr1-14713012221118441] Abreu W. Rodrigues T. Sequeira C. Pires R. Sanhudo A. (2018). The experience of psychological distress in family caregivers of people with dementia: A cross-sectional study. Perspectives in Psychiatric Care, 54(2), 317–323. 10.1111/ppc.12240 29077985

[bibr3-14713012221118441] Ally Y. (2009). Witch hunts in modern South Africa: An under-represented facet of gender- based violence. Medical Research Council, University of South Africa. http://www.mrc.ac.za/crime.crime (Accessed March 20, 2019).

[bibr4-14713012221118441] Alzheimer’s Association (2017). Alzheimer’s disease facts and figures. Alzheimer’s Dementia, 13(4), 325–373.

[bibr5-14713012221118441] Basu I. Mukhopadhyay S. (2019). Factors related to adverse mental health condition of demented family caregivers: A study in West Bengal, India. Anthropological Review, 82(4), 373–388. 10.2478/anre-2019-0028

[bibr6-14713012221118441] Bjørge H. Sæteren B. Ulstein I. D. (2019). Experience of companionship among family caregivers of persons with dementia: A qualitative study. Dementia, 18(1), 228–244. 10.1177/1471301216666172 27578815

[bibr7-14713012221118441] Black B. S. Johnston D. Rabins P. V. Morrison A. Lyketsos C. Samus Q. M. (2013). Unmet needs of community-residing persons with dementia and their informal caregivers: Findings from the MIND at Home Study. Journal of the American Geriatrics Society, 61(12), 2087–2095. 10.1111/jgs.12549 24479141 PMC4001885

[bibr8-14713012221118441] Blay S. L. Toledo Pisa P. E. (2010). Public stigma: The community’s tolerance of Alzheimer disease. American Journal of Geriatric Psychiatry, 18(2), 163–171. 10.1097/jgp.0b013e3181bea900 20104072

[bibr9-14713012221118441] Bosch J. N. (2014). The needs and experiences of caregivers of persons with Alzheimer’s Disease living in black rural communities in Mpumalanga. (Unpublished master’s thesis). University of Pretoria, South Africa.

[bibr10-14713012221118441] Braun V. Clarke V. (2006). Using thematic analysis in psychology. Qualitative Research in Psychology, 3(2), 77–101. 10.1191/1478088706qp063oa

[bibr11-14713012221118441] Braun V. Clarke V. (2019). Reflecting on reflexive thematic analysis. Qualitative Research in Sport, Exercise and Health, 11(4), 589–597. 10.1080/2159676X.2019.1628806

[bibr12-14713012221118441] Brodaty H. Donkin M. (2009). Family caregivers of people with dementia. Dialogues of Clinical Neuroscience, 11(2), 217–228. 10.31887/dcns.2009.11.2/hbrodaty PMC3181916 19585957

[bibr13-14713012221118441] Brooke J. Ojo O. (2019). Contemporary views on dementia as witchcraft in sub-Saharan Africa: A systematic literature review. Journal of Clinical Nursing, 29(Issues 1–2), 20–30. Blackwell Publishing Ltd. 10.1111/jocn.15066 31531993

[bibr14-14713012221118441] Bunn F. Goodman C. Sworn K. Rait G. Brayne C. Robinson L. McNeilly E. Steve Iliff S. (2012). Psychosocial factors that Shape patient and carer experiences of dementia diagnosis and treatment: A systematic review of qualitative studies. PLOS Medicine, 9(10), 1–12. 10.1371/journal.pmed.1001331 PMC3484131 23118618

[bibr15-14713012221118441] Byrne D. (2021). A worked example of Braun and Clarke’s approach to reflexive thematic analysis. Quality and Quantity, 56(2), 1–22. 10.1007/s11135-021-01182-y

[bibr16-14713012221118441] Chan E. Y. Phang K. N. Glass G. F. Lim W. S. (2019). Crossing, trudging and Settling: A phenomenological inquiry into lived experience of asian family caregivers of older persons with dementia. Geriatric Nursing, 40(5), 502–509. 10.1016/j.gerinurse.2019.03.015 30979516

[bibr17-14713012221118441] Cheng S. T. Lam L. C. Chan L. C. Law A. C. Fung A. W. Chan W. C. Tam C. W. Chan W. M. (2011). The effects of exposure to scenarios about dementia on stigma and attitudes toward dementia care in a Chinese community. International Psychogeriatrics, 23(9), 1433–1441. 10.1017/s1041610211000834 21729424

[bibr18-14713012221118441] Cheung D. S. K. Ho K. H. M. Cheung T. F. Lam S. C. Tse M. M. Y. (2018). Anticipatory grief of spousal and adult children caregivers of people with dementia 11 medical and health Sciences 1117 public health and health services 11 medical and health Sciences 1103 clinical Sciences. BMC Palliative Care, 17(1), 124. 10.1186/s12904-018-0376-3 30458746 PMC6247750

[bibr19-14713012221118441] Cipriani G. Borin E. (2014). Understanding dementia in the sociocultural context: A review. International Journal of Social Psychiatry, 61(2), 1–7. 10.1177/0020764014560357 25431401

[bibr20-14713012221118441] De Jager C. A. Joska J. A. Hoffman M. Borochowitz K. E. Combrink M. I. (2015). Dementia in rural South Africa: A pressing need for epidemiological studies. South African Medical Journal, 105(3), 189–190. 10.7196/SAMJ.8904 26294824

[bibr21-14713012221118441] De Jager C. A Msemburi W. Pepper K. Combrink I. (2017). Dementia prevalence in a rural region of South Africa: A cross-sectional community study. Journal of Alzheimer’s Disease, 60(3), 1087–1096. 10.3233/jad-170325 PMC5676974 28984589

[bibr22-14713012221118441] Devinsky O. D’esposito M. (2004). Neurology of cognitive and behavioural disorders. Oxford University Press.

[bibr23-14713012221118441] Doka K. (2004). Grief and dementia. In Doka K. (Ed.), Living with grief: Alzheimer’s disease (pp. 169–195). Hospice Foundation of America.

[bibr24-14713012221118441] Dong S. Duan Y. Hu Y. Zhao Z. (2012). Advances in the pathogenesis of Alzheimer’s disease: A re- evaluation of amyloid cascade hypothesis. Translational Neurodegeneration, 1(18), 1–12. 10.1186/2047-9158-1-18 23210692 PMC3526416

[bibr25-14713012221118441] Ducharme F. Kergoat M. J. Antoine P. Pasquier F. Coulombe R. (2013). The unique experience of spouses in early-onset dementia. American Journal of Alzheimer’s Disease and Other Dementias, 28(6), 634–641. 10.1177/1533317513494443 PMC10852552 23823140

[bibr27-14713012221118441] Fernando S. M. Deane F. P. McLeod H. J. (2010). Sri Lankan doctors’ and medical undergraduates’ attitudes towards mental illness. Social Psychiatry and Psychiatric Epidemiology, 45(7), 733–739. 10.1007/s00127-009-0113-6 19688283

[bibr28-14713012221118441] Ferreira M. Kalula S. (2009). Ageing, women and health: Emerging caregiving needs in sub-Saharan African countries. BOLD, 19(4), 2–12.

[bibr29-14713012221118441] Ferreira M. Makoni S. B. (2002). Towards a cultural and linguistic construction of late- life dementia in an urban African population. In Makoni S. B. Stroeken K. (Eds.), Ageing in Africa. Sociolinguistic and anthropological approaches (pp. 21–42). Ashgate.

[bibr30-14713012221118441] Fife B. Brooks-Cleator L. Lewis J. P. (2020). The world was shifting under our feet, so I turned to my devotionals as his dementia worsened”: The role of spirituality as a coping mechanism for family caregivers of Alaska native elders with dementia. Journal of Religion, Spirituality and Aging, 33(3), 252–270. 10.1080/15528030.2020.1754995

[bibr31-14713012221118441] Forbat L. (2003). Concepts and understandings of dementia by ‘gatekeepers’ and minority ethnic ‘service users. Journal of Health Psychology, 8(5), 645–655. 10.1177/13591053030085013 19177723

[bibr32-14713012221118441] GBD 2019 Collaborators. (2021). Global mortality from dementia: Application of a new method and results from the global burden of disease study 2019. Alzheimer’s Dementia: Translational Research & Clinical Interventions, 7(1), 1–28. 10.1002/trc2.12200 PMC8315276 34337138

[bibr33-14713012221118441] Gomez-Gomez C. Riquelme-Heras H. Aranda-Garza I. Gutierrez-Herrera R. Mendez- Espinosa E. Martinez-Lazcano F. Gutierrez- Sanchez P. (2018). Relationship of stigma to caregivers burden in Alzheimer’s disease patients. Journal of Primary Health Care and General Practice, 2(2), 1–7

[bibr34-14713012221118441] Graham N. Lindesay J. Katona C. Bertolote J. M. Camus V. Copeland J. R. M. Wancata J. de Mendonca Lima C. A. Gaillard M. Gely Nargeot M. C. Gray J. Jacobsson L. Kingma M. Kuhne N. O'Loughlin A. Rutz W. Saraceno B. Wancata J. (2003). Reducing stigma and discrimination against older people with mental disorders: A technical consensus statement. International Journal of Geriatric Psychiatry, 18(8), 670–678. 10.1002/gps.876 12891632

[bibr35-14713012221118441] Gurayah T. (2015). Caregiving for people with dementia in a rural context in South Africa. South African Family Practice, 57(3), 194–197. 10.1080/20786190.2014.976946

[bibr36-14713012221118441] Han J. W. Jeong H. Park J. Y Kim T. H. Lee D. Y. Lee D. W. Ryu S. H. Kim S. K. Yoon J. C. Jhoo J. Kim J. L. Lee S. B. Lee J. J. Kwak K. P. Kim B. J. Kim K. W. (2014). Effects of social supports on burden in caregivers of people with dementia. International Psychogeriatrics, 26(10), 1639–1648. 10.1017/s1041610214001331 25006855

[bibr37-14713012221118441] Harmell A. L. Chattillion E. A. Roepke S. K. Mausbach B. T. (2011). A review of the psychobiology of dementia caregiving: A focus on resilience factors. Current Psychiatry.Reports, 13(3), 219–224. 10.1007/s11920-011-0187-1 21312008 PMC3182821

[bibr38-14713012221118441] Hendriks-Lalla A. Pretorius C. (2018). The male familial caregiver experience of caring for persons with Alzheimer’s disease from low socioeconomic status: A South African perspective. Dementia, 0(0), 1–22. 10.1177/1471301218781372 29909650

[bibr39-14713012221118441] Hindley G. Kissima J. Oates L. L. Paddick S. M. Kisoli A. Brandsma C. Gray W. K. Walker R. W. Mushi D. Dotchin C. L. (2016). The role of traditional and faith healers in the treatment of dementia in Tanzania and the potential for collaboration with allopathic healthcare services. Age and Ageing, 46(1), 130–137. 10.1093/ageing/afw167 28181644

[bibr40-14713012221118441] Holley C. K. Mast B. T. (2009). The impact of anticipatory grief on caregiver burden in Dementia caregivers. Gerontologist, 49(3), 388–396. 10.1093/geront/gnp061 19386826

[bibr41-14713012221118441] Hooyman N. R. Kiyak H. A. (2002). Social Gerontology. A multidisciplinary perspective (6th ed.). Allyn and Bacon.

[bibr42-14713012221118441] Hossain M. Crossland J. Stores R. Dewey A. Hakak Y. (2020). Awareness and understanding of dementia in South Asians: A synthesis of qualitative evidence. Dementia, 19(5), 1441–1473. 10.1177/1471301218800641 30296834

[bibr43-14713012221118441] Hovland C. (2018). Welcoming death: Exploring pre-death grief experiences of caregivers of older Adults with dementia. Journal of Social Work in End-of-Life and Palliative Care, 14(4), 274–290. 10.1080/15524256.2018.1508538 30457443

[bibr44-14713012221118441] Hussain A. (2001). Islamic beliefs and mental healthJackson, DCompleting a PhD by publication: A review of Australian policy and implications for practice. Mental Health NursingHigher Education Research & Development, 2132(23), 6355–9368. 10.1080/07294360.2012.692666

[bibr144-14713012221118441] Jarrot S. E. Zarit S. H. Stephens M. A. P. Townsend A. Greene R. (2005). Instrumental help and caregivers\x{2019} distress: Effects of change in informal and formal help. American Journal of Alzheimer\x{2019}s Disease and Other Dementias, 20(3), 181–190. 10.1177/153331750502000308 PMC10833266 16003934

[bibr45-14713012221118441] Jett K. F. (2006). Mind-loss in the African American community: Dementia as a normal part of aging. Journal of Aging Studies, 20(1), 1–10. 10.1016/j.jaging.2005.05.002

[bibr46-14713012221118441] Joubert J. D. (2005). A profile of informal caregivers in South Africa. (Unpublished master’s thesis). University of Pretoria.

[bibr47-14713012221118441] Kabir Z. N. Leung A. Y. M. Grundberg Å. Boström A. M. Lämås K. Kallström A. P. Moberg C. Cronfalk B. S. Meijer S. Konradsen H. (2020). Care of family caregivers of persons with dementia (CaFCa) through a tailor-made mobile app: Study protocol of a complex intervention study. BMC Geriatrics, 20(1), 305. 10.1186/s12877-020-01712-7 32847495 PMC7449058

[bibr48-14713012221118441] Kahn P. V. Wishart H. A. Randolph J. S. Santulli R. B. (2016). Caregiver stigma and burden in memory disorders: An evaluation of the effects of caregiver type and gender. Current Gerontology and Geriatrics Research, 1, 1–5. 10.1155/2016/8316045 PMC4749763 26941795

[bibr49-14713012221118441] Kalula S. (2013). Medicine in the elderly: Unique challenges and management. Continuing Medical Education, 31(10), 352. http://cmej.org.za/index.php/cmej/article/view/2878/3234

[bibr50-14713012221118441] Kalula S. Petros G. (2011). Responses to dementia in less developed countries with a focus on South Africa. Global Aging, 7(1), 31–40.

[bibr51-14713012221118441] Kehoua G. Dubreuil C. M. Ndamba-Bandzouzi B. Guerchet M. Mbelesso P. Dartigues J. F. Preux P. M. (2019). People with dementia in sub-Saharan Africa: From support to abuse by caregivers: Results of EPIDEMCA-FU program in Congo. Dementia and Geriatric Cognitive Disorders Extra, 9(1), 163–175. 10.1159/000489846 31097954 PMC6489057

[bibr52-14713012221118441] Khonje V. Milligan C. Yako Y. Mabelane M. Borochowitz K. E. de Jager C. A. (2015). Knowledge, attitudes and beliefs about dementia in an urban Xhosa-speaking community in South Africa. Advances in Alzheimer’s Disease, 04(02), 21–36. 10.4236/aad.2015.42004

[bibr53-14713012221118441] Kobiske K. R. Bekhet A. K. Garnier-Villarreal M. Frenn M. (n.d.). Caregivers of partners with young-onset dementia caregivers of partners with young-onset dementia recommended citation recommended citation. SAGE Publications. https://epublications.marquette.edu/nursing_fac/585 10.1177/0193945918806689 30343648

[bibr54-14713012221118441] Krutter S. Schaffler-Schaden D. Essl-Maurer R. Wurm L. Seymer A. Kriechmayr C. Mann E. Osterbrink J. Flamm M. (2020). Comparing perspectives of family caregivers and healthcare professionals regarding caregiver burden in dementia care: Results of a mixed methods study in a rural setting. Age and Ageing, 49(2), 199–207. 10.1093/ageing/afz165 31875879 PMC7047818

[bibr55-14713012221118441] Leff D. (2014). Witch-hunts in South-Africa. Advocacy against human rights abuses committed as a result of accusations of witchcraft and violent witch-hunts. South African Pagan Rights Alliance. http://www.mediaforjustice.net/962/. (Accessed March 22, 2019).

[bibr56-14713012221118441] Lezak M. D. Howieson D. B. Bigler E. D. Tranel D. (2012). Neuropsychological assessment (5th ed.). Oxford University Press.

[bibr57-14713012221118441] Lietz C. A. Langer C. L. Furman R. (2006). Establishing trustworthiness in qualitative research in social work: Implications from a study regarding spirituality. Qualitative Social Work: Research and Practice, 5(4), 441–458. 10.1177/1473325006070288

[bibr58-14713012221118441] Lindauer A. Harvath T. A. (2014). Pre-death grief in the context of dementia caregiving: A concept analysis. Journal of Advanced Nursing, 70(10), 2196–2207. 10.1111/jan.12411 24702153

[bibr59-14713012221118441] Lindeza P. Rodrigues M. Costa J. Guerreiro M. Rosa M. M. (2020). Impact of dementia on informal care: A systematic review of family caregivers’ perceptions. In BMJ supportive and Palliative care. BMJ Publishing Group. 10.1136/bmjspcare-2020-002242 33055092

[bibr60-14713012221118441] Long T. Johnson M. (2000). Rigour, reliability and validity in qualitative research. Clinical Effectiveness in Nursing, 4(1), 30–37. 10.1054/cein.2000.0106

[bibr61-14713012221118441] Mackenzie J. (2006). Stigma and dementia: East European and South Asian family carers negotiating stigma in the U.K. Journal of Social Research Practice, 5(2), 233–247. 10.1177/1471301206062252

[bibr62-14713012221118441] MacLeod A. Tatangelo G. McCabe M. You E. (2017). There isn’t an easy way of finding the help that’s available. Barriers and facilitators of service use among dementia family caregivers: A qualitative study. International Psychogeriatrics, 29(5), 765–776. 10.1017/S1041610216002532 28351450

[bibr63-14713012221118441] Mahomed A. Pretorius C. (2020). Availability and utilization of support services for South African male caregivers of people with Alzheimer’s Disease in low-income communities. Dementia, 20(2), 633–652. 10.1177/1471301220909281 32138542

[bibr64-14713012221118441] Meyer J. C. Harirari P. Schellack N. (2016). Overview of Alzheimer’s disease and its management. South African Pharmacy Journal, 83(9), 48–56

[bibr65-14713012221118441] Meyer O. L. Nguyen K. H. Dao T. N. Vu P. Arean P. Hinton L. (2015). The sociocultural context of caregiving experiences for Vietnamese dementia family caregivers. Asian American Journal of Psychology, 6(3), 263–272. 10.1037/aap0000024 26617956 PMC4659380

[bibr66-14713012221118441] Mkhonto F. Hanssen I. (2018). When people with dementia are perceived as witches. Consequences for patients and nurse education in South Africa. Journal of Clinical Nursing, 27(1–2), Article e169–e176. 10.1111/jocn.13909 28557051

[bibr67-14713012221118441] Moore K. J. Crawley S. Vickerstaff V. Cooper C. King M. Sampson E. L. (2020). Is preparation for end of life associated with pre-death grief in caregivers of people with dementia? International Psychogeriatrics, 32(6), 753–763. 10.1017/S1041610220000289 32241317 PMC7612630

[bibr68-14713012221118441] Morgan D. Innes A. Kosteniuk J. (2011). Dementia care in rural and remote settings: A systematic review of formal or paid care. Maturitas, 68(1), 17–33. 10.1016/j.maturitas.2010.09.008 21041045

[bibr69-14713012221118441] Mugumbate J. Nyanguru A. (2013). Exploring African philosophy: The value of Ubuntu social work. African Journal of Social Work, 3(1), 82–100.

[bibr70-14713012221118441] Mukadam N. Cooper C. Livingston G. (2011). A systematic review of ethnicity and pathways to care in dementia. International Journal of Geriatric Psychiatry, 26(1), 12–20. 10.1002/gps.2484 21157846

[bibr71-14713012221118441] Mukadam N. Livingston G. (2012). Reducing the stigma associated with dementia: Approaches and goals. Aging Health, 8(4), 377–386. 10.2217/ahe.12.42

[bibr72-14713012221118441] Mushi D. Rongai A. Paddick S.-M. Dotchin C. Mtuya C. Walker R. (2014). Social representation and practices related to dementia in Hai District of Tanzania. BMC Public Health, 14(2014), 260. http://www.biomedcentral.com/1471-2458/14/260 24642112 10.1186/1471-2458-14-260 PMC3994576

[bibr73-14713012221118441] Navab E. Negarandeh R. Peyrovi H. (2012). Lived experiences of Iranian family member caregivers of persons with Alzheimer’s disease: Caring as “captured in the whirlpool of time. Journal of Clinical Nursing, 21(7–8), 1078–1086. 10.1111/j.1365-2702.2011.03960.x 22289075

[bibr74-14713012221118441] Nichols E. Szoeke C. E. Vollset S. E. Abbasi N. Abd-Allah F. Abdela J. Aichour M. T. E. O’Akinyemi R. Alahdab F. Asgedom S. W. Awasthi A. Barker-Collo S. L. Baune B. T. Béjot Y. Belachew A. B. Bennett D. A. Biadgo B. Bijani A. Bin Sayeed M. S. Brayne C. (2019). Global, regional, and national burden of Alzheimer’s disease and other dementias, 1990–2016: A systematic analysis for the global burden of disease study 2016. Lancet Neurology, 18(1), 88–106. 10.1016/S1474-4422(18)30403-4 30497964 PMC6291454

[bibr75-14713012221118441] Omiya T. Kutsumi M. Fukui S. (2021). Work, leisure time activities, and mental health among family caregivers of the elder people in Japan. Healthcare, 9(2), 129. 10.3390/healthcare9020129 33525664 PMC7911960

[bibr76-14713012221118441] Owokuhaisa J. Rukundo G. Z. Wakida E. Obua C. Buss S. S. (2020). Community perceptions about dementia in southwestern Uganda. BMC Geriatrics, 20(1), 135. 10.1186/s12877-020-01543-6 32293301 PMC7158106

[bibr77-14713012221118441] Passoni S. AlessioToraldo A. Villa B. Bottini G. (2015). Prolonged grief in caregivers of community-dwelling dementia patients. American Journal of Alzheimer’s Disease & Other Dementias, 30(2), 192–200. 10.1177/1533317514542643 PMC10852694 25013118

[bibr78-14713012221118441] Perry-Young L. Owen G. Kelly S. Owens C. (2018). How people come to recognise a problem and seek medical help for a person showing early signs of dementia: A systematic review and meta-ethnography. Dementia, 17(1), 34–60. 10.1177/1471301215626889 26764265 PMC5758935

[bibr80-14713012221118441] Polenick C. A. Struble L. M. Stanislawski B. Turnwald M. Broderick B. Gitlin L. N. Kales H. C. (2020). I’ve learned to just go with the flow”: Family caregivers’ strategies for managing behavioral and psychological symptoms of dementia. Dementia, 19(3), 590–605. 10.1177/1471301218780768 29886777 PMC6522324

[bibr81-14713012221118441] Pretorius C. Walker S. Heyns P. (2009). Sense of coherence among male caregivers in dementia: A South African perspective. Dementia, 8(1), 79–84. 10.1177/1471301208099046

[bibr82-14713012221118441] Prorok J. C. Horgan S Seitz D. P. (2013). Health care experiences of people with dementia and their caregivers: A meta-ethnographic analysis of qualitative studies. CMAJ, 185(14), 1–12. 10.1503/cmaj.121795 PMC3787191 24003093

[bibr83-14713012221118441] Ransmayr G. Hermann P. Sallinger K. Benke T Seiler S. Dal-BiancoSchmidt P. R. Marksteiner J. Defrancesco M. Sanin G. Struhal W. Guger M. Vosko M. Hagenauer K. Lehner R. Futschik A. (2018). Caregiving and caregiver burden in dementia home care: Results from the prospective dementia registry (PRODEM) of the Austrian Alzheimer Society. Journal of Alzheimer’s Disease, 63(1), 103–114. 10.3233/jad-170657 29614643

[bibr84-14713012221118441] Robinson K. M. Buckwalter K. C. Reed D. (2005). Predictors of use of services among dementia caregivers. Western Journal of Nursing Research, 27(2), 126–140. 10.1177/0193945904272453 15695566

[bibr85-14713012221118441] Sanders S. Ott C. H. Kelber S. T. Noonan P. (2008). The experience of high levels of grief. In caregivers of persons with Alzheimer’s disease and related dementia. Death Studies, 32(6), 495–523. 10.1080/07481180802138845 18958942

[bibr86-14713012221118441] SAPRA [South African Pagan Rights Alliance] (2015). Remember their names – victims of witch-hunts in South Africa 2000–2015. www.paganrightsalliance.org (Accessed March 22, 2019).

[bibr87-14713012221118441] Scheba A. Turok I. N. (2019). Strengthening township economies in South Africa: The case for better regulation and policy innovation. Urban Forum, 31(1), 77–94. 10.1007/s12132-019-09378-0

[bibr88-14713012221118441] Schüz B. Czerniawski A. Davie N. Miller L. Quinn M. G. King C. Carr A. Elliott K. E. J. Robinson A. Scott J. L. (2015). Leisure time activities and mental health in informal dementia caregivers. Applied Psychology: Health and Well-Being, 7(2), 230–248. 10.1111/aphw.12046 26097155

[bibr89-14713012221118441] Slattum P. W. Peron E. P. Hill A. (2014). Alzheimer's disease. In DiPiro J. T. Talbert R. L. Yee G. C. Matzke G. R. Wells B. G. Posey L. (Eds.), Pharmacotherapy: A Pathophysiologic approach (pp. 817–834). McGraw-Hill.

[bibr91-14713012221118441] Song J. A. Park M. Park J. Cheon H. J. Lee M. (2018). Patient and caregiver interplay in behavioral and psychological symptoms of dementia: Family caregiver’s experience. Clinical Nursing Research, 27(1), 12–34. 10.1177/1054773816678979 27864478

[bibr92-14713012221118441] Spigelmyer P. C. Hupcey J. E. Smith C. A. Loeb S. J. Kitko L. (2018). Resistiveness to care as experienced by family caregivers providing care for someone with dementia. Journal of Nursing Scholarship, 50(1), 36–46. 10.1111/jnu.12345 28914991

[bibr93-14713012221118441] Statistics South Africa (2012). Statistical release. Census 2011. Statistics South Africa. http://www.statssa.gov.za/publications/p03014/p030142011.pdf

[bibr94-14713012221118441] Su J. A. Chang C. C. (2020). Association between family caregiver burden and affiliate stigma in the families of people with dementia. International Journal of Environmental Research and Public Health, 17(8), 2772. 10.3390/ijerph17082772 PMC7215659 32316454

[bibr95-14713012221118441] Takai M. Takahashi M. Iwamitsu Y. Oishi S. Miyaoka H. (2011). Subjective experiences of family caregivers of patients with dementia as predictive factors of quality of life. Psychogeriatrics, 11(2), 98–104. 10.1111/j.1479-8301.2011.00354.x 21707857

[bibr96-14713012221118441] Teahan Á. Lafferty A. Fealy G. McAuliffe E. Phelan A. O’Sullivan L. O’Shea D. (2016). 163AN evidence synthesis of resilience in dementia caregiving: A systematic review. Age and Ageing, 45(suppl 2), ii1–ii12. 10.1093/ageing/afw159.31

[bibr97-14713012221118441] Tremont G. (2011). Family caregiving in dementia. Medicine and Health, 94(2), 36–38. PMC3487163 21456372

[bibr98-14713012221118441] Truzzi A. Valente L. Ulstein I. Engelhardt E. Laks J. Engedal K. (2012). Burnout in familial caregivers of patients with dementia. Revista Brasileira de Psiquiatria, 34(4), 405–412. 10.1016/j.rbp.2012.02.006 23429811

[bibr99-14713012221118441] Tuomola J. Soon J. Fisher P. Yap P. (2016). Lived experience of caregivers of persons with dementia and the impact on their sense of self: A qualitative study in Singapore. Journal of Cross-Cultural Gerontology, 31(2), 157–172. 10.1007/s10823-016-9287-z 26923465

[bibr100-14713012221118441] Vallée A. Vallée J. N. (2017). Ressenti des aidants de personnes âgées dépendantes atteintes de démence. In Geriatrie et Psychologie Neuropsychiatrie du Vieillissement, 15(Issue 2), 138–144. John Libbey Eurotext. 10.1684/pnv.2017.0663 28625933

[bibr102-14713012221118441] Van der Poel R. Pretorius C. (2009). Dementia in low and middle income countries: The need for research and advocacy. Dementia, 8(4), 451–454. 10.1177/1471301209350290 (Accessed March 9, 2019).

[bibr145-14713012221118441] Van Wijngaarden E. Van der Wedden H. Henning Z. Komen R. The A.-M. (2018). Entangled in uncertainty: The experience of living with dementia from the perspective of family caregivers. PLoS ONE, 13(6), 1–21. 10.1371/journal.pone.0198034 PMC5999274 29897922

[bibr104-14713012221118441] Visser M. Moleko A. G. (2012). Community psychology in South Africa (2nd ed.). Van Schaik Publishers.

[bibr105-14713012221118441] Willig C. (2008). Introducing qualitative research in psychology (2nd ed.). Bell and Bain Ltd.

[bibr106-14713012221118441] Win K. K. Chong M. S. Ali N. Chan M. Lim W. S. (2017). Burden among family caregivers of dementia in the oldest-old: An exploratory study. Frontiers in Medicine, 4(NOV), 205. 10.3389/fmed.2017.00205 29204426 PMC5698684

[bibr107-14713012221118441] World Health Organization (2021). Global status report on the public health response to dementia. World Health Organization.

[bibr108-14713012221118441] Xiong C. Biscardi M. Astell A. Nalder E. Cameron J. I. Mihailidis A. Colantonio A. (2020). Sex and gender differences in caregiving burden experienced by family caregivers of persons with dementia: A systematic review. PLoS ONE, 15(4), Article e0231848. 10.1371/journal.pone.0231848 32310969 PMC7170244

[bibr109-14713012221118441] Yang F. Ran M. Luo W. (2019). Depression of persons with dementia and family caregiver burden: Finding positives in caregiving as a moderator. Geriatrics and Gerontology International, 19(5), 414–418. 10.1111/ggi.13632 30773779

[bibr110-14713012221118441] Yap L. K. P. Seow C. C. D. Henderson L. M. Goh Y. N. J. (2005). Family caregivers and caregiving in dementia. Reviews in Clinical Gerontology, 15(3–4), 263–271. 10.1017/S0959259806001900

