# Supplementary material for: Understanding the lived experiences of family caregivers of individuals with dementia in Soweto, a South African Township
Source: Dementia (London). 2022 Aug 13;21(7):2264–87. doi: 10.1177/14713012221118441 (PMC9606014; doi:10.1177/14713012221118441)
Supplement: Supplemental Material - Understanding the lived experiences of family caregivers of individuals with dementia in Soweto, a South African Township [file sj-pdf-1-dem-10.1177_14713012221118441.pdf]

Supplemental Material: Comprehensive overview of themes and illustrative quotes

Broad Theme	Subtheme	Illustrative Quotes
**Theme 1: Understanding Dementia**	*Perceptions*	"My culture they think that it's a black magic or there is sometimes it's the ancestors" (Q1)<br>"My culture? Some people said it is witchcraft you know because some, like my mother will go out, go to the next door and see things in her house. So they said it is witchcraft. You see" (Q2)<br>"In my culture… you find that there are aunties, old ladies who've been banned, who've been characterized no, who've been called witches and they either they were killed, they were stoned, they were banned because they bewitch others and others will be going in the open naked and then they will be branded witches yes" (Q3)<br>"I think us in the more urban areas we know they call it like an old people's disease. So they don't really define it as dementia. They just say aaah it's normal. She's old" (Q4)<br>My understanding is that people, I think they lose, I don't know, they are too forgetful" (Q5)<br>"It's just like she doesn't remember. So, and it's not like she's sick, you know" (Q6)<br>"The way I make sense of it is it is what it is. People get sick, others have cancer and my mum has dementia or others have TB or others have diabetes. It just is what it is" (Q7)<br>"I think Dementia is another illness that what it just happens or it's hereditary" (Q8)<br>"So I mean about me, I know this is a disease" (Q9)<br>"I don't think we know it, it's just people are crazy, they're going mad. I guess that's the blanket term that will be used" (Q10)<br>"Others do not understand because like maybe they think that hey, this person she is crazy. This person - he is crazy" (Q11)<br>"Ja before from people who actually who told us like she is… I think… insane" (Q12)<br>"You know when my mom is coming, if she's angry, she will bang the door, bang the gate, everything, going, passing through all the people and if you are angry it's not easy for you. You know we were like a laughing stock. But now people, as they know now she is, she has a dementia. They appreciate, you know, and they understand" (Q13)
	Challenges	"I came here 2016 but I was like I don't understand why, why, why this person have to be like this" (Q14)<br>"You know what? Before, before I did not understand it, yes, I was like that, because of I used to shout at her even. Why doing this? Why are you doing that?" (Q15)<br>"From reading, you know what I did in 2016 I took her to one of our local clinics and I said to the doctor it was for something else but I asked the doctor to help me I said this is what I see my mom is doing, this she's going through this, she say don't you know it is old age but then my mother knew that there was such an illness you know and the doctors just let me go dismissed me and just said it is old age." (Q16)<br>"I think at first, because we didn't know. So in the beginning, because we didn't know, we were left to our assumptions. Because obviously, even though we had questions, but we're also African. So when you go to places where medical practitioners are supposed to know, and then they're saying they don't know, but now we know that they didn't know because they were not psychiatrists or mental doctors in a sense" (Q17)<br>"Umm the challenges were… you know if a person doesn't say I've got a pain here, I've got a pain here. You just keep quiet and then you see that he's just changing and then there's something in the body or he might say this is painful or my heart or my headache? That was my real challenge that. How would we help

	her? Because now you take the person to the hospital and when they ask , because they ask you know what is happening? What is wrong? Then what I'm going to say? Because this person just quiet. He doesn't say anything. The pain or… you see. So that was my real challenge" (Q18)<br>Oh no I just figured out but you know he needs something but where do I get some help. Where to go. Who will understand what is eating my husband because I told the Sister's and the clinic just didn't know. They just though that he is getting old. And now how do I explain to the other Doctor about what is going on with him? (Q19)<br>"Okay. That's when we say "okay, she's not okay" and then we went to the Clinic but still it took time because you know clinics, they didn't like transfer us to the doctor that she is in right now. It took us surely a year doing the same thing over and over again. Just to get treatment after being diagnosed, just to get treatment"(Q20)<br>"I think at the time the community also did not understand that she was sick, because at some point she would take her card and give it to that one – can you please keep it for me because you know, that child is stealing my money and whatever. Until they, until someone went to them looking for that card and whatever and told them no, no, this person – how can her child steal the money from her while she's the one doing things for her?" (Q21)<br>"Yes because I was tired not knowing what to do, because if it was so worse this time, because she was fighting and going outside and crying to other people, people they will come to me and say your mother is always crying. She is saying that you don't take care of her, and I would say I don't understand. And then your mother say that your children they, they are, they give her a problem. So it becomes something that now, it's like, we are treating her bad. And people now are becoming involved you know. And me I was trying, telling myself that one day she is going to be all right as long as we can keep on doing what is right towards her, but no. Nothing had helped, nothing, nothing you can do whatever you know to do, you can try to understand but you will never understand her. So I saw it becoming worse now as she is going outside crying to other people, it is worse. So I have just decided that I should take the police and take her to hospital." (Q22)
Enduring Ambiguity	"To be serious … to be honest, I don't understand dementia" (Q23)<br>"I don't know, maybe she has some irrational fear that it's contagious or I don't know" (Q24)<br>"Look, how I make sense of them is – you see my daughter over here – one thing I'm seeing is that there are habits in how we remember and we recall and remember anyway. And I see those habits in us, all of us, so I think it's, I don't want to call I dementia, but it's just a habit in recalling and thinking. So I've just seen that with her it's gotten to like a scary place, I'll call it that" (Q25)<br>"People just say that it is old age, right? But I do not even know how to explain it. I just think that it is old age as well" (Q26)<br>"I've googled all kinds of things. But what I understand is the, it's basically the brain sort of being, I don't know how to describe it but slowly but surely the cells get destroyed, something like that. And that's why you start seeing early signs and then it progresses to other things and they end up becoming people that are completely, you need to now lock them in, you need to … they cannot, they cannot be on their own" (Q27)<br>"Because since she got Alzheimer's we don't know what's happening in her brain and this time she is worse. And now she's worse, worse. I don't know she's maybe in which stage. I know there's 5 stages, I don't know. You tell her something now, 2 seconds she forgot. 2 Seconds she forgot" (Q28)<br>"I learnt a lot more through the talk, let me put it that way" (29)

**Theme 2: Struggles and Sacrifice**	*Personal Sacrifices*	“So, I am no longer working. I just told myself I need to take care of my mother” (Q30)<br>“Yes, so at the end of the first term I left work, I had to” (Q31)<br>“Well I had to, I had to leave my job five years ago decide ja to say I’ll stay with this woman to understand what is it that she’s going through before I can bring in someone you know who would be our help yes” (Q32)<br>“Like I said you know, when you haven’t been able to meet your goals in life as a person, I’m a very ambitious individual. I’m a go getter, I want things done and I drive them. I’ve worked a lot in community doing community work and facilitated how to do my own business and run an NGO, you know big goals and all that and not being able to should I say achieve or do that, you know has been… I’ve taken a knock out of that” (Q33)<br>“I was saying this year I’m going, I’m definitely going to Thailand for a couple of weeks. And now, that’s not happening. And even though I still want plan to go next year I’m thinking even those just two weeks that I want to go for, you know, are… Is she going to take her medication? Is she going to eat? I’m worried about all those things” (Q34)
	Caregiver Roles	“But now you've got to kind of be a parent to Mom also” (Q35)<br>“Whenever I took decisions, she will fight with me saying that I want to be the mother of the house. She is the mother it is so difficult” (Q36)<br>“And when my Dad comes, she will be like … he will be like "oh why didn't you cook and you know I am hungry?” I'm like "I'm taking care of Mom and I'm taking care of my son. Sometimes I get tired" (Q37)<br>“Yes, it's because I have children, of which I have to take care of, and eish, yes, as I am single mother, as I am a single mother I need to ensure that my kids, I have to put bread on top of the table nè, yes, and taking care of my mother of which we had ups and downs and no one would understand my problem” (Q38)<br>“So, you are taking care of mom? You are taking care of the household? Taking care of your siblings? If you do not take care of yourself? You feel like there is no choice?” (Q39)<br>“Maybe the other thing that is stressing me is my granddaughter she has got, she is epilepsy, she forget a lot and she has got symptoms similar to my husband, because you ask her something, I have forgotten, I have forgotten. Yes maybe that is why I stressing so much yes” (Q40)
	Lack of Support	“Keeping clean yes. The teeth, you know, such things. Yes…so… so, I did that. I did that together with my son because my son did realise: “Mom, I think this is so stressful” (Q41)<br>“What made it difficult it was when I’m alone and nobody is around to help me” (Q42)<br>“My twin sister she lives at her husband’s house and the other one she’s got her own house at the Glen, so they don’t want to help me – I’m alone. They don’t want to come there and sleep with my mother or maybe take my mother to their places, they don’t want – it’s me alone, I must look, I must take care” (Q43)<br>“But sometimes I can feel it because there is nobody to help me. You understand? I am alone. I have to clean. Make sure the house is clean. To cook, to wash, everything is upon my shoulders” (Q44)<br>“There, actually there’s a lot of support, but as I’m saying we, we aren’t seeing things eye to eye, so that’s the problem. There’s a lot of love, there’s a lot of concern, there’s a lot of care” (Q45)<br>“So that’s that, but the family from my father’s side, I think they like to think they’re being supportive, but sometimes they speak things that are hurtful to us. I’m not sure if they’re not aware that these things are

		hurtful or they intend to, yes” (Q46)<br>“People are not supportive, they just listen to your stuff, that’s the support you get, they just listen, they don’t give you advice on what you should do, how to handle the person what not to do you see, they don’t give you that on how to take care of the situation when it happens, so I won’t say I’ve had support from the outside that much no” (Q47)
	Social Withdrawal	“I’m forever sending excuses, I can’t attend funerals, weddings, parties, family things – I’m always putting excuses” (Q48)<br>“So… But I’ve been going out less since she was diagnosed with Alzheimer’s. Again, it comes back to that guilt thing of I’m out here having fun and she’s at home maybe bored or something” (Q49)<br>“I'm not sure because we're always inside these days. I don't even go out like I use to because I don't feel like.. what if something happen when I'm out? You know” (Q50)<br>Like my life has changed, I’m always at home. I’m no more the person who’ll go and socialize with people because like today when I’m off, I know that I’ll have to clean and do everything and go and see my friends, now I cannot do that. I have to be with, I’m always at home, like now when I finish here I have to rush home” (Q51)<br>“So now I’m also consciously avoiding other people because I’m avoiding that encounter of me telling them, and what sucks is when I meet those people, the first thing they say, oh, how’s your mum, I’m like, uh, she’s okay, like ja, but she’ll get better, I’m like okay, she will, but then no” (Q52)<br>“I wouldn’t go, even when we go the short distance to the families, I must have my gloves, I must have my 2 diapers spare, I must have my wipes, I must have my disposable bags, should we be there for 2, 3 hours, you’re sitting there because you can’t leave him alone. I must carry all those things. And when people go up and down chatting, chatting, I must keep on checking up on him. But no, let me go and check – it’s stressful” (Q53)
**Theme 3: Mental Health**	*Psychological Distress*	“Just constantly thinking about it. Constantly thinking about it, constantly worrying about it and thinking about the unknown. How far is it going to go with her, is she going to do the things that we heard that other people do, how long does she have before she leaves us. You know, you just ask yourself about the future that is not yet here, and it’s very, it can drain you mentally” (Q54)<br>“It makes me feel sad, and fearful at the same time, why fearful because this are times when this when this disease has escalated, there are times there are times when they disappear, so I’m just thinking what will happen, when will that happen, you know” (Q55)<br>“I was sad and scared at the same time because you know, when you are asleep and someone, you wake up and someone is standing at your bed, you don’t know what they are going to do. What if one day it is something that is dangerous and maybe a knife or something and she cuts me you know? I was scared, I was scared, I don’t want to lie. And when she start her moods I will just go into my bedroom and keep quiet and say that I am not going to answer, I’m not going to say anything, I’m just not going to answer” (Q56)<br>“It’s anxiety more than anything, and it’s anxiety – to be very honest – it’s the anxiety around being able to control the environment. Ja, that’s my thing. Like when I feel it’s outside of my ability to handle, that’s when I get very anxious” (Q57)<br><br>“You do not know what to do…but you get frustrated because you do not know what to do” (Q58)<br>“You just go with her. With the feeling of that moment of whether whatever you are saying or whatever you

		are trying is going to make her feel better but you do not know. So, it is so frustrating knowing that if you had possibly a solution then you would know that if I did this then but you do not know. So that is how frustrating that is" (Q59)<br>"Because to be honest, these, the people with dementia, the kind of behaviours that they have sometimes, it's overwhelming" (Q60)<br>"Ja. I didn't accept that easily, because you sort of like wanted to… you said, ag, maybe it's not dementia, maybe it was just something she forgot. You know, stuff like that" (Q61)<br>… "it's not easy, it's not easy for me really to accept that this is really my mom going through this, it's heart-breaking, it's heart-breaking." (Q62)<br>"When you try to communicate with them and then they don't respond. So if you don't understand, it will be like … I don't know, you'll be angry" (Q63)<br>"It is, it does get heavy. Like I find I'm very – and she picks it up as well – I'm very, I get very irritable" (Q64)<br>"Well, before I found out about Alzheimer's I say, it was extremely stressful for me also" (Q65)
	Caregiver grief	"So it's very difficult to lose someone while you are still looking at them – they are here" (Q66)<br>"So you're grieving the person they used to be because she's definitely not the same person she was before. And you're grieving them in your lives because, you know, they're going to eventually stop talking. They're going to forget who you are. You are used to watching them slip away every day" (Q67)<br>"He is no longer the man that I got married to. He is someone else" (Q68)<br>"He was a good, like I told you, he was a good father. He can't do all those things that he used to do for us as a family, and we miss him, yet he is there, and we miss the things he used to do for us" (Q69)<br>"I always say to people this is worse than death because when a person passes on, you know there's closure, you bury them and then you deal with the process in terms of grief, so in my case, I'm grieving the loss of my mother even though she's here" (Q70)<br>"Just knowing that I'm actually saying goodbye to her, so she deteriorating by the day and there's nothing I can do about it" (Q71)
**Theme 4: Protective Factors**	*Coping Mechanisms*	"I think it's because of prayers all the time that I become so strong" (Q72)<br>"It is very difficult. I pray. If I feel, even if my emotions are just down, I just sit down and pray. God, please make understand my mother. Make me, you know because if I cannot, who is going to take care of her? Other people who will not understand her the way I do understand. So, I pray. Yes" (Q73)<br>"Music, it just calms me down" (Q74)<br>"Yes so I think that's how I calm myself down because if I feel that my mind has started and I don't understand myself, I usually go the library and then read some books that that are interesting or maybe there is a subject that I want to know about it, the time for me to take that book and sit down and read and take newspaper round and read, that's how I calm myself down" (Q75)
	Loyalty, Devotion and Commitment	"I really want to take care of my mother, it's not just lip service" (Q76)<br>"You just tell yourself that it is my mom. I cannot throw her on the street or take her to a home. It is worse there because if I am frustrated what about the people at her room. Maybe they deal with her differently. Ja, so I rather be the one who is frustrated about her with all the family members" (Q77)<br>"Even if I have a helper sometimes it gets frustrating 'cos you feel like they don't know enough to help you. It's better to do things yourself. That's why I ended up coming to stay with mum, because I found that, you

		know, there's little things but they just don't do it properly. And it just felt like maybe it's better if I go and do it myself" (Q78)<br>"So this one is directed to me – he's my husband I must carry him where they cannot afford to help" (Q79)
	Strengthened Bonds	"…it makes me to love her more. And funny enough, even if she says those things in my absence, when I knock at the door, she's like, she opens her arms, she wants to hug me and I would, okay, and I would give her a hug and tell her that I love her and I need her and whatever. So, it makes me understand the disease and not the person so that I love her regardless of what she is doing" (Q80)<br>"So it changed me, because, before I knew the disease, I couldn't understand, I couldn't. But now it makes me to love her because I know that she's sick" (Q81)<br>"The most fun part about it you see, because now sometimes she's a good mood, she talks about stuff, the olden day stuff, that they used to do that I feel it's fine I'm connecting with my granny, I'm actually bonding with her, where it feels good though, ja those are the fun parts when she's excited to you know" (Q82)
	Developing Resilience and Personal Growth	"Now, I think I am kind of getting used to that thing that like he has dementia and needs to be taken care of because he forgets very quick. So, right now I am getting used to it. Yes. I am fine. I think I have adapted to this thing, yes" (Q83)<br>"Yes, with the family. We just take it as it comes and just make sure that we try and make it better for everyone" (Q84)<br>"Sometimes you sleep through the noise. You just get used to it. If this is what you know, there's not much, your mind adjusts to it. Yeah, your mind somehow adjusts and you are able to live, it's not ideal but that's what it is" (Q85)<br>"I must acclimate myself to the situation" (Q86)<br>"What helped me not to change is because I felt yes, in life, things will happen to you and if you want to be okay, accept that so that I can help this person who is now like this" (Q87)<br>"So you need to be patient and understand that they don't understand the feeling that you are feeling. They don't understand what's going on. To them it's just normal" (Q88)<br>"I have learnt you have to be patient and you have to have an open heart or ja those are the two basic things you have to have you see" (Q89)<br>"Oh, I have learned so much. I had to grow very patient, loving… You know you need to be very strong and you need to understand people" (Q90)
